# A systematic review and meta-analysis of the efficacy and safety of glucocorticoids in the treatment of severe pneumonia

**DOI:** 10.1016/j.clinsp.2025.100630

**Published:** 2025-04-23

**Authors:** Jingye Liu, Zhiqiang Yang

**Affiliations:** a EICU, Zhoushan Hospital, Zhejiang, China; b Department of Respiratory and Critical Care Medicine, Zhoushan Hospital, Zhejiang, China

**Keywords:** Glucocorticoids, Severe pneumonia, Serum CRP, Meta-analysis

## Abstract

**Objectives:**

To systematically evaluate the efficacy and superiority of glucocorticoids in the treatment of severe pneumonia.

**Method:**

Randomized Controlled Trials (RCTs) on glucocorticoids in the treatment of severe pneumonia were retrieved from CNKI, CBM, China Science and Technology Journal Database (VIP), Wanfang Database, and PubMed as of January 1, 2017. The literature was independently and objectively screened, extracted, and evaluated by two researchers, and a meta-analysis of the extracted data was performed using Revman 5.3 software.

**Results:**

Ten studies that met the inclusion criteria were included, with a cumulative total of 1120 cases. The meta-analysis results confirmed that the observation group was superior to the control group in terms of efficacy rate, temperature recovery time, cough relief time, rale disappearance time, and serum CRP level. There was no statistically significant difference in terms of the incidence of adverse reactions, mortality rate, and reinfection rate between the two groups.

**Conclusions:**

Glucocorticoids showed an obvious clinical efficacy in patients with severe pneumonia. However, due to the small number of included studies and the ambiguity of numerous bias risk assessments, high-quality and large-sample RCTs are still needed to provide corroborating evidence.

## Highlights

• Glucocorticoids could effectively improve the condition of patients with severe pneumonia.
• Glucocorticoids could effectively shorten the temperature recovery time, cough relief time, and rale disappearance time.
• Glucocorticoids could effectively reduce the length of stay, and regulate the serum CRP level.

## Introduction

Severe pneumonia is one of the most common critical respiratory conditions in clinical practice. Its pathogenic factors often involve exposure to single or multiple potent pathogens or drug-resistant bacteria infections. Moreover, its severity is closely associated with the degree of local inflammation and its dissemination. Epidemiological studies^1^^,^^2^ have shown that the common pathogenic bacteria are Staphylococcus aureus and Streptococcus pneumoniae, and the most common drug-resistant bacteria are methicillin-resistant Staphylococcus aureus. In recent years, there has been an increase in severe pneumonia cases. The condition of the disease progresses rapidly and can easily develop into sepsis and multiple organ failure, among which the inflammation caused by multiple organ failure is the main prognostic risk factor, affecting the prognosis and development of the disease.^3^ The clinical manifestations of patients with severe pneumonia include high fever, chills, dyspnea, etc., and in severe cases, abnormality of consciousness and septic shock may occur.^4^ Studies have shown that the case fatality rate of severe pneumonia is as high as 30 % to 50 %. Severe pneumonia is more likely to occur in the elderly and children, both of whom have weaker resistance. Elderly patients often experience degenerative organ changes, and decreased immune levels, and frequently have underlying diseases. As a result, their illnesses tend to progress more dangerously and critically, with rapid disease progression. Without timely treatment, it can progress to toxic or shock pneumonia, posing considerable treatment challenges.^5^ Therefore, effective and accurate treatment of severe pneumonia is extremely important. Glucocorticoids are commonly used in clinical practice to treat severe pneumonia, possessing anti-inflammatory and important immunomodulatory effects, and many studies suggest that glucocorticoids are widely used in the treatment of severe pneumonia, as they can reduce inflammatory cell infiltration, phagocytosis, and capillary dilation in the initial stages of inflammation. During the severe inflammation stage, glucocorticoids can inhibit fibroblasts and their excessive proliferation, which is often the cause of fibrosis.^6^ Therefore, glucocorticoids are also widely used in clinical settings for the treatment of severe pneumonia.

## Materials and methods

### Literature inclusion criteria

The object of study: Patients clinically diagnosed with severe pneumonia.

Intervention measures: The intervention measures of the observation group were glucocorticoid therapy, and the control group received other therapies.

Outcome indicators: The evaluation indicators included total efficacy rate, incidence of adverse reactions, mortality rate, reinfection rate, temperature recovery time, cough relief time, rale disappearance time, serum CRP, PaO2 increase time, serum IL-6, serum TNF-α, length of stay, PEF, FEV1, PCT, asthma disappearance time, and oxygenation index.

Study type: Randomized Controlled Trials (RCTs) of glucocorticoid therapy in patients with severe pneumonia. Ethical approval was waived because this article does not contain any studies with human or animal subjects performed by any of the authors.

### Literature exclusion criteria

This study aimed to investigate the efficacy of glucocorticoid therapy in patients with severe pneumonia. Consequently, it excluded review, literature research, summary of experiences, theoretical discussion, and other types of non-randomized controlled trials; animal experiments; duplication of literature; trials addressing severe pneumonia treatment without glucocorticoid intervention; RCTs involving glucocorticoid treatment combined with other therapies, as such interventions could affect the determination of the outcomes; case report; clinical studies with missing outcomes.

### Literature retrieval

China National Knowledge Internet (CNKI), China Science and Technology Journal Database (VIP), Wanfang database, Chinese BioMedical Literature Database (CBM), PubMed and other electronic databases were searched, and the Chinese keywords were “糖皮质激素, 重症肺炎, 随机对照”; The English keywords were “Glucocorticoids, severe pneumonia, randomized controlled”. The search date was January 2017 as of June 15, 2023.

### Data extraction and quality evaluation

Two researchers completed the literature search and data extraction independently and objectively based on the inclusion and exclusion criteria of the study, paid attention to the preservation of original objective data, and designed the data extraction form in advance to implement the pre-extraction, which included details such as the names of the authors, publication year, sample size, randomization methods, intervention approaches, study outcomes, and information about attrition or drop out. In case of disagreement, data should be checked and processed with the third researcher. In terms of quality evaluation, the Cochrane risk of bias tool was utilized to evaluate the risk of bias, including selection bias (generation of random sequences, concealed allocation), performance bias (blinding of participants and researchers), detection bias (blinding of outcome assessments), attrition bias (incomplete outcome observation data), reporting bias (selective reporting), and other biases.

### Statistical methods

Using Revman 5.3 software, statistical analysis was conducted according to the principles of experimental medicine, and the continuous variables such as temperature recovery time, cough relief time, rale disappearance time, serum CRP, PaO2 increase time, serum IL-6, serum TNF-α, length of stay, PEF, FEV1, PCT, asthma disappearance time, and oxygenation index were combined using the Weighted Mean Difference (WMD). According to the combined effect and 95 % CI, heterogeneity was examined using *Q* test and *I^2^* test. When *p* ≥ 0.01 and *I^2^* < 50 %, it indicated homogeneity in statistical significance between the two groups, and in such cases, a fixed-effect model was employed for the combination. Conversely, it indicated high heterogeneity and a random-effects model was employed in the combination. Subgroup analysis and sensitivity analysis were applied to explore the influence of grouping factors on the results and to investigate the sources of heterogeneity from a statistical perspective.

## Results

### Literature screening results

**Fig. 1:**
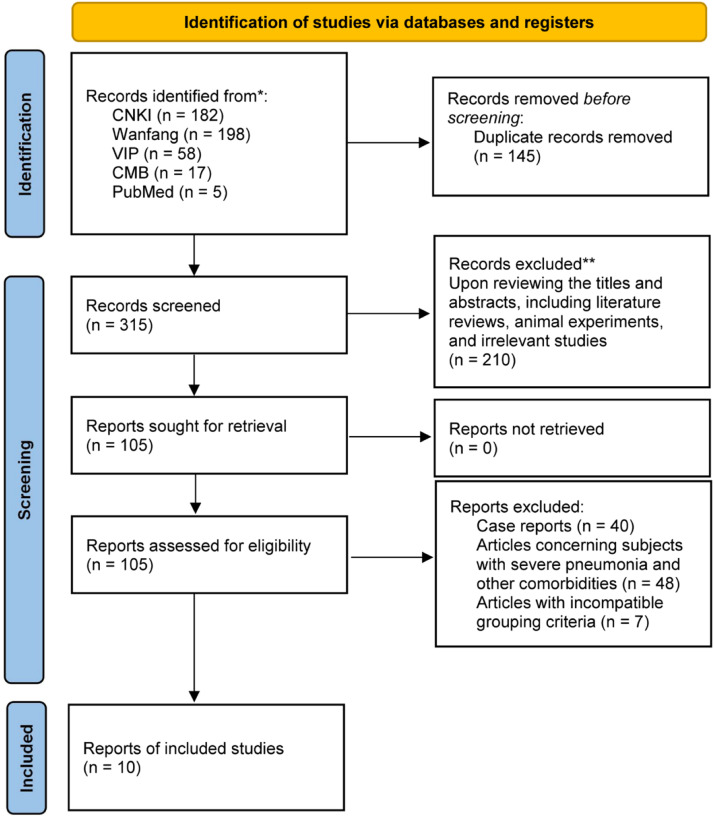
Flow diagram of included papers.

After searching CNKI, VIP, Wanfang database, CBM, and PubMed, 460 articles were retrieved. Among them, 145 duplicate articles were removed. Upon reviewing the titles and abstracts, a total of 210 articles were excluded, including literature reviews, animal experiments, theoretical explorations, bibliometric studies, interventions combined with other therapies, and irrelevant research papers. The remaining 105 articles underwent further meticulous examination of their full texts, leading to the exclusion of 95 articles (comprising 40 case reports, 48 articles concerning subjects with severe pneumonia and other comorbidities, and 7 articles with incompatible grouping criteria). Eventually, 10 articles were considered eligible and included in the study, as indicated in Fig. 1.

Basic characteristics of the included studies

Among the 10 articles included, there were a total of 1120 patients with severe pneumonia, as indicated in Table 1.

**Table 1:** Basic characteristics of the included studies.

Included articles	Sample size (O/C)	Patient type	Intervention measures		Course of treatment	Outcome indicators
			Observation group	Control group		
Sun JH et al. 2021^7^	40/40	Pediatric	Glucocorticoid	Conventional therapy	4 weeks	①②⑧⑩⑪
Peng P et al. 2020^8^	60/60	Adult	Glucocorticoid	Conventional therapy	10 days	①②⑤⑥⑦⑧⑨⑩⑪⑮
Xu JW 2019^9^	75/75	Pediatric	Glucocorticoid	Conventional therapy	3 weeks	①②⑤⑥⑧⑩⑪⑫
Xu YH et al. 2018^10^	34/34	Adult	Glucocorticoid	Conventional therapy	7 days	①⑤⑫⑮⑯
Li F 2017^11^	36/36	Adult	Glucocorticoid	Conventional therapy	‒	③④⑤⑦⑨⑫
Li JL 2020^12^	41/41	Adult	Glucocorticoid	Conventional therapy	1 week	①②③④⑥⑦⑧⑨⑫⑯
Chu ZD et al. 2018^13^	50/50	Adult	Glucocorticoid	Conventional therapy	1 week	①④⑧⑫
Chen QF et al. 2023^14^	65/65	Pediatric	Glucocorticoid	Conventional therapy	4 weeks	①②⑤⑥⑦⑧⑫⑬⑭
Han Y et al. 2020^15^	50/50	Pediatric	Glucocorticoid	Conventional therapy	7 days	①②⑩⑪⑬⑭
Huang SF 2017^16^	109/109	Pediatric	Glucocorticoid	Conventional therapy	7 days	①②⑤⑥⑦⑧⑫

*Note*: O, Observation group; C, Control group. ① Total efficacy rate, ② Incidence of adverse reactions, ③ Mortality rate, ④ Reinfection rate, ⑤ Temperature recovery time, ⑥ Cough relief time, ⑦ Rale disappearance time, ⑧ Serum CRP, ⑨ PaO2 increase time, ⑩ Serum IL-6, ⑪ Serum TNF-α, ⑫ Length of stay, ⑬ PEF, ⑭ FEV1, ⑮ Asthma disappearance time, ⑯ Oxygenation index.

### Methodological quality evaluation

**Fig. 2:**
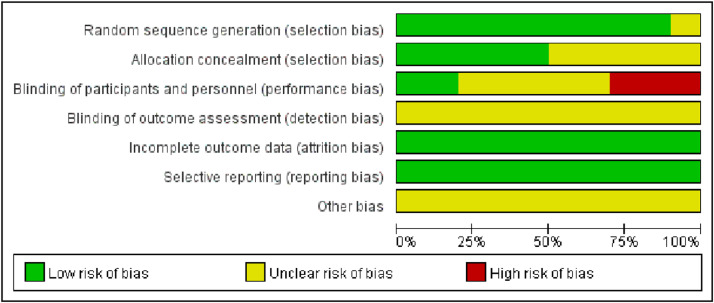
Risk of bias analysis chart.

**Fig. 3:**
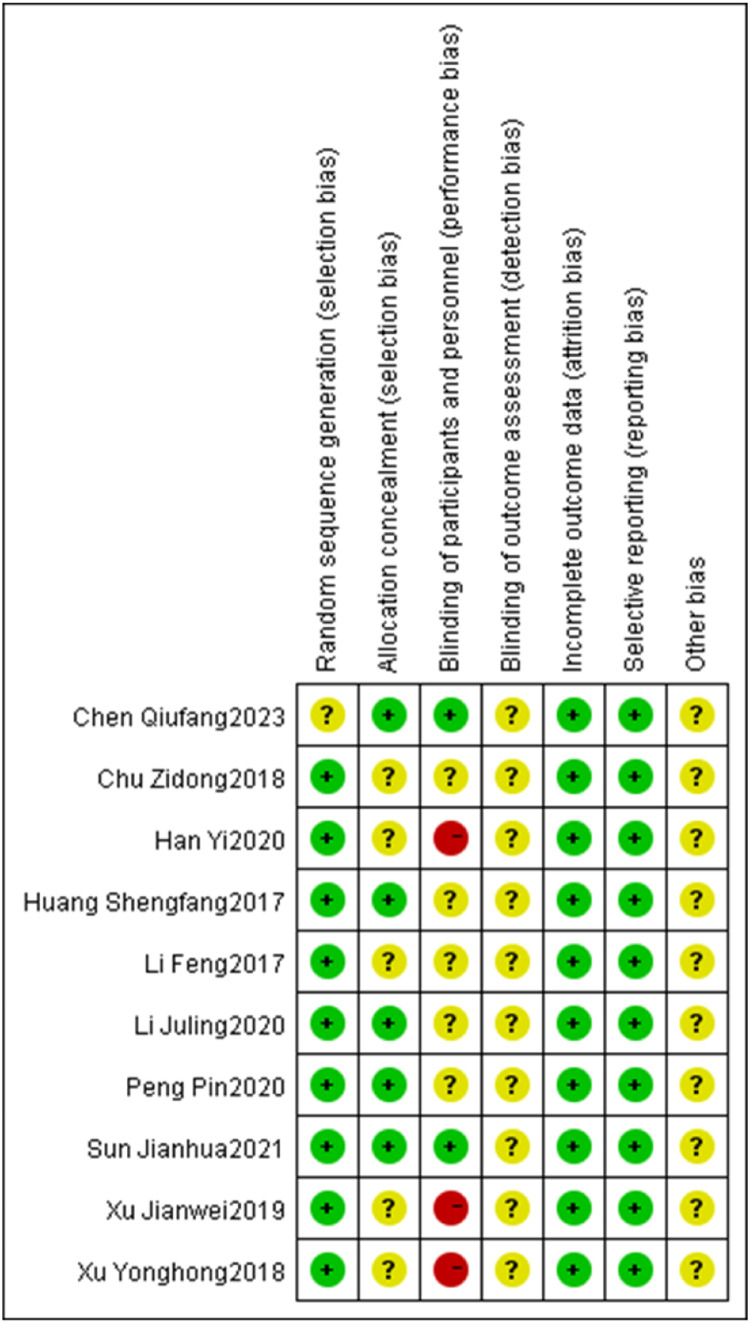
Percentage risk of bias graph.

Of the 10 included studies, 5 referred to the method of random number table allocation, thus evaluating them as low risk in the random allocation method item. On the other hand, 3 studies were evaluated as high risk in the blinding evaluation item. Due to the difficulty of implementing blinding during the intervention, only two studies mentioned implementing blinding and the remaining studies were judged to have unclear risks (Figs. 2, 3).

### Meta-analysis results

#### Total efficacy rate

**Fig. 4:**
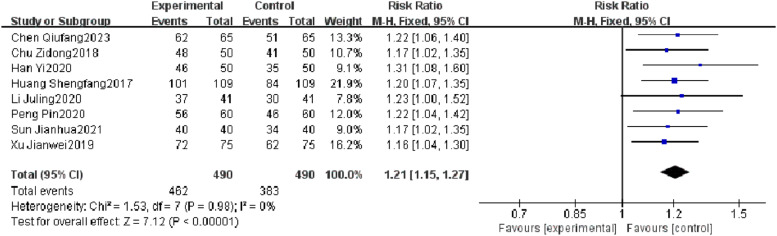
Comparison of total efficacy rate between observation group and control group.

Eight RCTs reported a total efficacy rate, and heterogeneity test results showed no significant heterogeneity (*p* = 0.98, *I^2^* = 0 %). The results showed that the total efficacy rate of the observation group was significantly different from that of the control group (*I^2^* = 0 %, RR = 1.21, 95 % CI [1.15, 1.27], *p* < 0.00001). The total efficacy rate of the observation group was superior to that of the control group (Fig. 4).

#### Incidence of adverse reactions

**Fig. 5:**
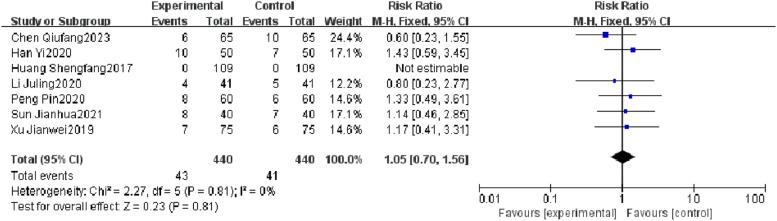
Comparison of incidence of adverse reactions between observation group and control group.

Seven RCTs reported an incidence of adverse reactions, and heterogeneity test results showed no significant heterogeneity (*p* = 0.81, *I^2^* = 0 %). There was no significant difference in the incidence of adverse reactions between the observation group and the control group (*I^2^* = 0 %, RR = 1.05, 95 % CI [0.70, 1.56], *p* = 0.81). The results showed that the incidence of adverse reactions of patients with severe pneumonia was not effectively improved in the observation group (Fig. 5).

#### Mortality rate

**Fig. 6:**
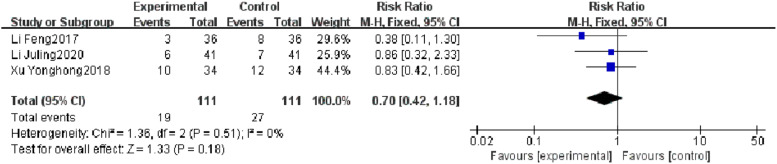
Comparison of mortality rate between observation group and control group.

Three RCTs reported a mortality rate, and heterogeneity test results showed no significant heterogeneity (*p* = 0.18, *I^2^* = 0 %). There was no significant difference in mortality rate between the observation group and the control group (*I^2^* = 0 %, RR = 0.70, 95 % CI [0.42, 1.18], *p* = 0.18). The results showed that of the mortality rate of patients with severe pneumonia was not found to be reduced effectively in the observation group, which may be related to the limited number of included literature and the small sample size (Fig. 6).

#### Reinfection rate

**Fig. 7:**
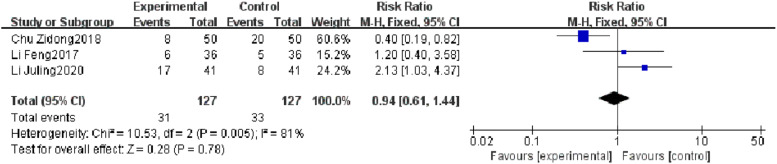
Comparison of reinfection rate between observation group and control group.

Three RCTs reported a reinfection rate, and heterogeneity test results showed significant heterogeneity (*p* = 0.005, *I^2^* = 81 %). Subgroup analysis was carried out according to the research quality and characteristics of research objects, and no source of heterogeneity was found. There was no significant difference in the reinfection rate between the observation group and the control group (*I^2^* = 81 %, RR = 0.94, 95 % CI [0.61, 1.44], *p* = 0.78). The results showed that the reinfection rate of patients with severe pneumonia was not found to be reduced effectively in the observation group (Fig. 7).

#### Temperature recovery time

**Fig. 8:**
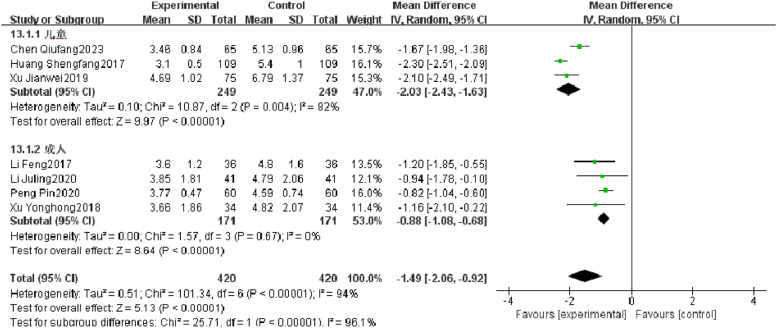
Comparison of temperature recovery time between observation group and control group.

Seven RCTs reported temperature recovery time of patients with severe pneumonia, and heterogeneity test results showed significant heterogeneity (*p* < 0.00001, *I^2^* = 94 %). Subgroup analysis was performed, and the results showed that there was no significant heterogeneity in improving the temperature recovery time in the observation group. After combination, it was found that the temperature recovery time of the observation group was significantly superior to that of the control group (*I^2^* = 96.1 %, MD = −1.49, 95 % CI [−2.06, −0.92], *p* < 0.00001) (Fig. 8).

#### Cough relief time

**Fig. 9:**
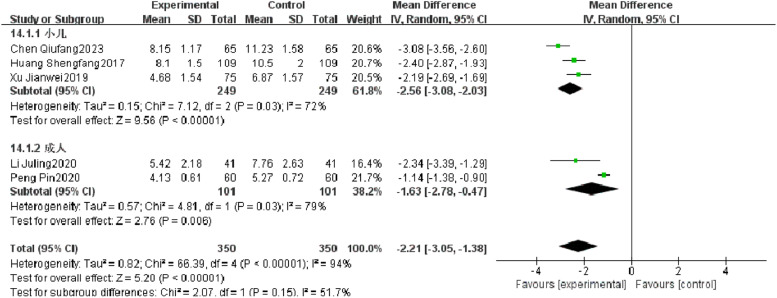
Comparison of cough relief time between observation group and control group.

Five RCTs reported the cough relief time of patients with severe pneumonia, and heterogeneity test results showed significant heterogeneity (*p* < 0.00001, *I^2^* = 94 %). Subgroup analysis was performed, and the results showed that the type of research object is not the source of heterogeneity. After combination, it was found that the observation group was significantly superior to the control group in reducing the cough relief time (*I^2^* = 94 %, MD = −2.21, 95 % CI [−3.05, −1.38], *p* < 0.00001) (Fig. 9).

#### Rale disappearance time

**Fig. 10:**
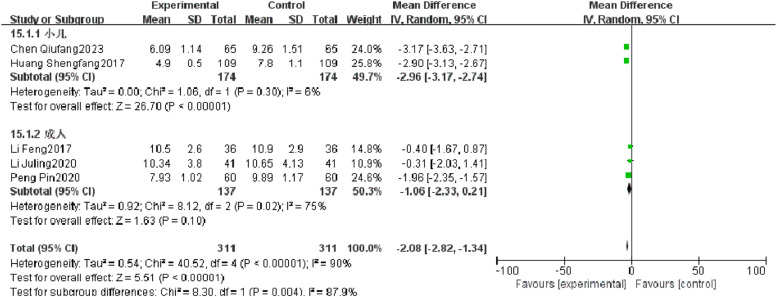
Comparison of rale disappearance time between observation group and control group.

Five RCTs reported rale disappearance time of patients with severe pneumonia, and heterogeneity test results showed significant heterogeneity (*p* < 0.00001, *I^2^* = 90 %). Subgroup analysis was performed, and the results showed that the type of research object may be the source of heterogeneity. After combination, it was found that the observation group was significantly superior to the control group in reducing the rale disappearance time (*I^2^* = 90 %, MD = −2.08, 95 %CI [−2.82, −1.34], *p* < 0.00001) (Fig. 10).

#### Serum CRP

**Fig. 11:**
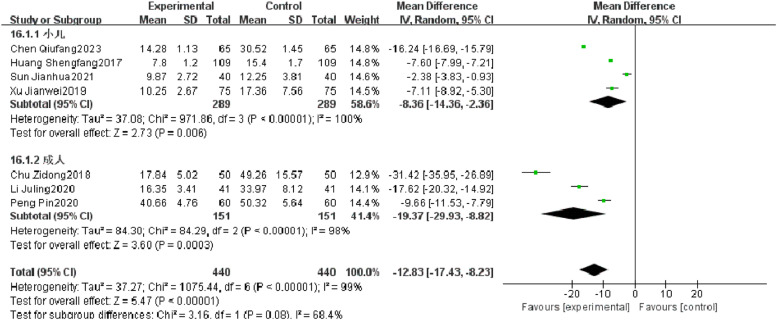
Comparison of serum CRP level between observation group and control group.

Seven RCTs reported serum CRP of patients with severe pneumonia, and heterogeneity test results showed significant heterogeneity (*p* < 0.00001, *I^2^* = 99 %). After subgroup analysis, high heterogeneity was found. After combination, it was found that the serum CRP level of the observation group was significantly superior to that of the control group (*I^2^* = 99 %, MD = −12.83, 95 % CI [−17.43, −8.23], *p* < 0.00001) (Fig. 11).

#### Length of stay

**Fig. 12:**
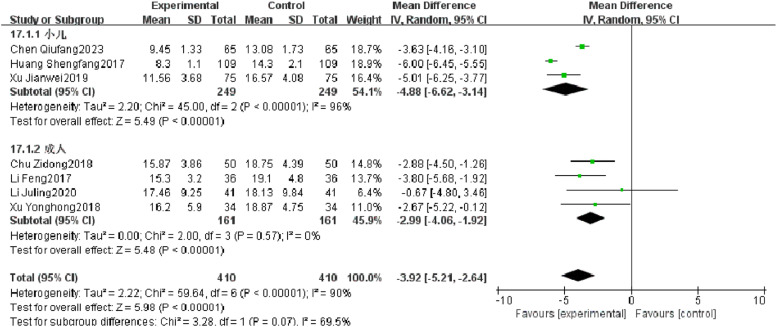
Comparison of length of stay between observation group and control group.

Seven RCTs reported length of stay of patients with severe pneumonia, and heterogeneity test results showed significant heterogeneity (*p* < 0.00001, *I^2^* = 90 %). Subgroup analysis was performed, and the results showed that there was no significant heterogeneity in length of stay in adults, but there was still high heterogeneity in length of stay in children. After combination, the observation group was significantly superior to the control group in reducing the length of stay (*I^2^* = 90 %, MD = −3.92, 95 % CI [−5.21, −2.64], *p* < 0.00001) (Fig. 12).

#### Publication bias

**Fig. 13:**
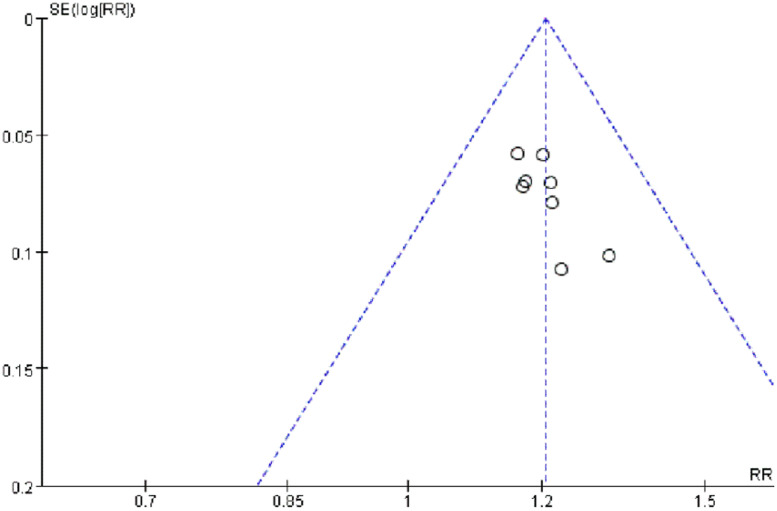
Funnel plot of efficacy rate in patients with severe pneumonia.

The bias analysis of the efficacy rate in patients with severe pneumonia was conducted. With the MD value of each study as the horizontal coordinate and the inverse of the log standard error of the MD value as the vertical coordinate, a funnel plot was plotted to assess publication bias among the included studies. The results showed that the graphs were basically symmetrical and normally distributed, suggesting that there was no certain degree of publication bias in the included literature (Fig. 13).

## Discussion

Severe pneumonia is one of the common respiratory critical illnesses in clinical practice. As a systemic disease, its main characteristics are systemic toxic reactions, respiratory system failure, etc. The condition of the disease progresses rapidly and is prone to sepsis and multiple organ dysfunction in the later stage, in which inflammation caused by the involvement of multiple organs, such as the nervous system, digestive system, and circulatory system dysfunction, is the main prognostic risk factor, and even leads to death. At this time, a safe and effective treatment plan is needed to alleviate the clinical symptoms caused by the disease and improve the cure rate.^6^

**Fig. 14:**
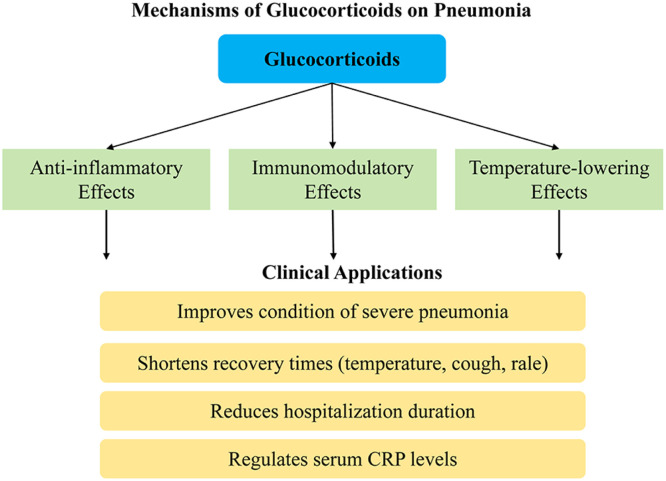
Central figure of mechanisms of glucocorticoids on the pneumonia.

Glucocorticoids have anti-inflammatory and immunomodulatory effects. Studies have indicated that it can effectively suppress inflammatory responses, such as the synthesis and release of inflammatory mediators, and inhibit the invasion of inflammatory cells into tissues, as well as modulate the function of lymphocytes and reduce immune impairment.^17^^,^^18^ After the glucocorticoids enter the bloodstream, they can readily bind to the endogenous albumin in the body, resulting in the formation of a complex capable of binding to small amounts of free glucocorticoids as receptors.^19^ As the glucocorticoids entering the body exert a temperature-lowering effect, they also have an inhibitory and alleviative effect on persistent inflammation in the body (Fig. 14).

The present study employed meta-analysis to include 10 clinical studies from both domestic and international sources, with a total of 1120 participants suffering from severe pneumonia, so to assess the efficacy and safety of glucocorticoids. The analysis results proved that glucocorticoids could effectively improve the condition of patients with severe pneumonia, effectively shorten the temperature recovery time, cough relief time, and rale disappearance time, reduce the length of stay, and regulate the serum CRP level. Numerous studies have confirmed that CRP, as the most widely used acute-phase inflammatory protein, rises rapidly when stimulated by inflammation, and that the value of its concentration in serum can reflect the development of the body's immune system, especially in cases of acute-phase infections, wherein the CRP levels in patients with inflammation or trauma are elevated in a short period of time.

The results of the meta-analysis confirmed that the observation group outperformed the control group in terms of efficacy, temperature recovery time, cough relief time, rale disappearance time, and serum CRP levels. There were no statistically significant differences in the incidence of adverse reactions, mortality rate, or reinfection rate. Consequently, it is concluded that glucocorticoids have a significant clinical effect on patients with severe pneumonia.

## Conclusion

This study provided a comprehensive comparison of the overall efficacy, incidence of adverse reaction, mortality rate, reinfection rate, temperature recovery time, cough relief time, rale disappearance time, and hospitalization duration of glucocorticoid treatment for severe pneumonia through a meta-analysis. Additionally, each outcome indicator was analyzed, offering robust clinical reference for the application of glucocorticoid therapy in severe pneumonia.

There are also some limitations to the results of this analysis. Firstly, the number of included literature is small. The language of literature retrieval was limited to Chinese and English, which largely resulted in the lack of literature inclusion, making the analysis results somewhat limited. Combined with the risk of bias assessment of the included studies, it showed that there was a high risk of bias in the literature. For example, in performance bias, it is difficult to achieve true blinding because, in actual clinical trials, one must consider not only the study design but also the current medical environment, and most subjects tend to be informed in advance about the procedures, intervention methods, and relevant precautions. Secondly, due to the inclusion of pediatric subjects in the literature, poor compliance is inevitable. At the same time, there are no definitive conclusions regarding the timing, optimal dosage, and best course of treatment for the use of glucocorticoids in severe pneumonia. Therefore, it is necessary to standardize the experimental design scheme in future RCTs to reduce the occurrence of various risk factors of bias. For the observation of outcome indicators, objective observational data such as inflammatory factors can be selected. Specific records should be made for attrition and adverse reactions to enhance the scientific rigor of the research. In the future, meta-analysis should incorporate larger sample sizes, multiple perspectives, and high-quality RCTs to offer a broader and more valuable array of treatment options for severe pneumonia in clinical settings.

## Funding

This research did not receive any specific grant from funding agencies in the public, commercial, or not-for-profit sectors.

## CRediT authorship contribution statement

Jingye Liu and Zhiqiang Yang designed experiments, carried out experiments, analyzed experimental results. Jingye Liu wrote the manuscript. Zhiqiang Yang revised the manuscript.

## Conflicts of interest

The authors declare no conflicts of interest.
